# Intramedullary Kirschner Wire Fixation for Metatarsal Fractures: A Comprehensive Review of Treatment Outcomes

**DOI:** 10.7759/cureus.59368

**Published:** 2024-04-30

**Authors:** Vatsal Patel, Sanjay V Deshpande, Sachin Goel, Anmol Suneja, Vivek H Jadawala

**Affiliations:** 1 Orthopedics, Jawaharlal Nehru Medical College, Datta Meghe Institute of Higher Education and Research, Wardha, IND

**Keywords:** patient selection, surgical technique, rehabilitation protocols, orthopedic surgery, intramedullary kirschner wire fixation, metatarsal fractures

## Abstract

Metatarsal fractures pose significant challenges in orthopedic practice, necessitating effective treatment methods to ensure optimal patient outcomes. This comprehensive review focuses on intramedullary Kirschner wire fixation as a promising intervention for metatarsal fractures. Beginning with an overview of metatarsal fractures and the imperative for effective treatments, the review delves into intramedullary fixation's definition, historical background, advantages, and disadvantages. Indications for its use in metatarsal fractures are discussed, providing a foundation for understanding its application. The surgical technique section outlines critical aspects, including patient selection criteria and preoperative planning. Before presenting a detailed step-by-step procedure for intramedullary Kirschner wire fixation, anesthesia considerations are explored. Emphasizing precision, fluoroscopic guidance, and meticulous postoperative care, this section provides insights for surgeons and healthcare practitioners. Considerations for rehabilitation follow, addressing postoperative care, expected recovery timelines, and physical therapy recommendations. Early mobilization, weight-bearing guidelines, and a structured rehabilitation program play pivotal roles in recovery. In the conclusion, key findings are summarized, highlighting the efficacy of intramedullary Kirschner wire fixation, its advantages, and recommendations for clinical practice. Additionally, areas for future research are identified, guiding further exploration and refinement of this surgical approach. This review is valuable for clinicians, researchers, and healthcare practitioners involved in metatarsal fracture management, contributing to the evolution of treatment strategies and improving patient care.

## Introduction and background

Metatarsal fractures, a common orthopedics challenge, pose significant implications for mobility and quality of life. This section provides a foundational understanding of these fractures, underscores the importance of efficacious treatment methods, and introduces the focus of this comprehensive review: intramedullary Kirschner wire (K-wire) fixation [1]. Metatarsal fractures, comprising a spectrum of injuries to the long bones of the foot, result from trauma, repetitive stress, or underlying medical conditions. These fractures vary in severity, including avulsion, stress, and displaced fractures. Understanding metatarsal fracture anatomy, biomechanics, and etiology is crucial for tailoring effective treatment strategies.

Effective management of metatarsal fractures is paramount to ensuring optimal functional outcomes and preventing long-term complications. Untreated or improperly managed fractures may lead to persistent pain, deformities, and impaired foot function. Therefore, exploring and implementing advanced treatment modalities is essential for achieving successful patient outcomes and mitigating the long-term impact of metatarsal fractures [2].

Among the evolving landscape of treatment options, intramedullary K-wire fixation has emerged as a promising intervention for metatarsal fractures. This technique involves the placement of a K-wire within the medullary canal of the metatarsal bones, providing stability and facilitating the healing process. As we delve into this method, we aim to comprehensively assess its efficacy, advantages, and potential limitations [3].

This review aims to critically examine the existing body of literature on intramedullary K-wire fixation for metatarsal fractures. By synthesizing information from clinical studies, case reports, and emerging research, we seek to provide a comprehensive overview of the treatment outcomes associated with this technique. Our goal is to offer valuable insights for clinicians, researchers, and healthcare practitioners involved in managing metatarsal fractures, ultimately contributing to enhanced patient care and improved clinical decision-making. By exploring surgical techniques, treatment outcomes, case studies, and future directions, this review aims to serve as a valuable resource for advancing the understanding and application of intramedullary K-wire fixation in the context of metatarsal fractures.

## Review

Overview of intramedullary K-wire fixation

Definition and Purpose

Intramedullary K-wire fixation involves the placement of K-wires within the medullary canal of the metatarsal bones, providing internal support and stability. The primary purpose of this surgical technique is to facilitate the alignment and healing of metatarsal fractures by immobilizing the affected bones, enabling optimal conditions for the natural healing process. The fixation aims to restore function, reduce pain, and prevent long-term complications associated with metatarsal fractures [4].

The roots of intramedullary fixation trace back to early orthopedic practices. Historical records reveal the utilization of rudimentary techniques for fracture stabilization, gradually evolving into more sophisticated methods over time. The advent of K-wires, named after the German surgeon Martin Kirschner, marked a significant advancement in orthopedic instrumentation. Since then, intramedullary K-wire fixation has undergone refinements, becoming a well-established and widely adopted approach in managing various fractures, including metatarsal fractures [5].

Advantages of Intramedullary Fixation

Stability: Intramedullary fixation offers inherent stability, a crucial factor in successfully managing metatarsal fractures. Securing K-wires within the medullary canal of the metatarsal bones minimizes the risk of malalignment during the healing process. The stability provided plays a pivotal role in facilitating anatomical restoration, ensuring that the fractured segments maintain proper alignment as the bone heals. This contributes to the functional recovery of the foot and reduces the likelihood of long-term complications associated with misaligned fractures [6].

Minimally invasive: One of the noteworthy advantages of intramedullary K-wire fixation is its relatively minimally invasive nature. Compared to other surgical interventions for metatarsal fractures, this technique involves smaller incisions and less disruption to surrounding tissues. The reduced invasiveness contributes to a more cosmetically favorable outcome and holds the potential for quicker recovery times. Patients undergoing minimally invasive procedures may experience less postoperative pain, reduced scarring, and a shorter recovery period, promoting overall patient satisfaction and a faster return to normal activities [7].

Early mobilization: The stability provided by intramedullary fixation enables patients to engage in early mobilization and weight-bearing exercises. Early mobilization is critical to rehabilitation, promoting joint flexibility, muscle strength, and overall functional recovery. With a stable internal fixation, patients can often begin controlled movements and weight-bearing activities sooner than with other treatment modalities. This early engagement in rehabilitation contributes to faster recovery times, reduces the risk of joint stiffness, and enhances the overall success of the treatment by facilitating a more rapid return to daily activities [8].

Reduced soft tissue trauma: Intramedullary fixation minimizes disruption to surrounding soft tissues compared to external fixation methods. The surgical technique involves accessing the metatarsal bone through a smaller incision, lessening trauma to the skin, muscles, and other soft tissues. This reduction in soft tissue trauma can lead to several benefits, including decreased postoperative pain, lower risk of wound complications, and a potentially more expedited recovery process. The preservation of soft tissue integrity is precious in the delicate structures of the foot, where minimizing trauma contributes to better surgical outcomes and patient comfort [9].

Disadvantages of Intramedullary Fixation

Risk of infection: Despite the benefits of intramedullary K-wire fixation, it is essential to acknowledge the inherent risk of infection associated with any surgical procedure. Maintaining strict aseptic techniques during surgery is paramount to minimize this risk. Inadequate sterilization, contamination of surgical instruments, or breaches in the surgical field can increase the likelihood of postoperative infections. Surgeons must adhere rigorously to established protocols, including proper preoperative skin preparation, sterile draping, and sterile instruments, to mitigate the risk of infection. Additionally, postoperative monitoring and prompt intervention in case of signs of infection are crucial for optimal patient outcomes [10].

Complications with hardware: While intramedullary K-wire fixation is generally considered a reliable method, complications related to the hardware may arise. Migration, breakage, or irritation from the implanted K-wires are potential issues that require consideration. Migration refers to the unintended movement of the wires from their initial placement, which can compromise stability and fracture alignment. Breakage of wires may occur due to factors such as excessive stress or inadequate fixation. Patient discomfort or irritation at the site of the implanted hardware is another complication that necessitates attention. Regular follow-up appointments with appropriate imaging studies are essential to identify and address hardware-related complications promptly. In some cases, revision surgery may be required to address these issues and ensure the continued efficacy of the fixation [11].

**Figure 1:**
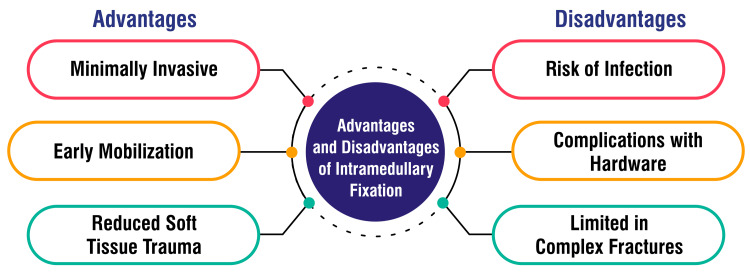
Advantages and disadvantages of intramedullary fixation The image is created by the corresponding author.

Limited in complex fractures: Intramedullary fixation, while effective for many metatarsal fractures, may have limitations in specific complex fracture patterns. The suitability of this technique depends on factors such as fracture location, configuration, and the extent of comminution. Alternative approaches may be more appropriate in cases where the fracture is highly complex, involving multiple metatarsals or intricate anatomical structures. Surgeons must carefully assess the specific characteristics of the fracture to determine the most suitable fixation method. This underscores the importance of individualized treatment plans, considering the unique aspects of each patient's injury. Advances in surgical techniques and technologies may contribute to expanding the applicability of intramedullary fixation in addressing a broader range of fracture complexities [2]. Figure 1 shows the advantages and disadvantages of intramedullary fixation.

Indications for Its Use in Metatarsal Fractures

Displaced fractures: Intramedullary fixation is particularly beneficial when dealing with metatarsal fractures that exhibit significant displacement. Displacement occurs when the fractured ends of the bone are no longer in proper alignment, posing challenges to the natural healing process. Intramedullary K-wire fixation is an effective intervention by realigning and stabilizing the fractured segments. When strategically placed within the medullary canal, the wires provide internal support, allowing for optimal anatomical restoration. By addressing displacement, this method helps mitigate the risk of long-term complications, such as joint deformities or impaired function, associated with improperly aligned fractures [2].

Unstable fractures: Intramedullary fixation is well-suited for fractures with instability. Unstable fractures often involve fragmented or displaced bone segments, posing a risk of further displacement during the healing phase. Intramedullary K-wires act as internal stabilizers, maintaining alignment and preventing excessive movement of the fractured segments. This stability is crucial for creating an environment conducive to the natural healing process, reducing the risk of complications such as malunion or nonunion. By providing internal support, intramedullary fixation contributes to restoring stability, fostering optimal conditions for fracture healing [12].

Delayed union or nonunion: Intramedullary fixation becomes a valuable therapeutic option when metatarsal fractures exhibit delayed union or nonunion. Delayed union refers to a slower-than-expected healing process, while nonunion denotes the failure of the fractured bones to heal. In such cases, the additional stability offered by intramedullary K-wires can promote and expedite the healing process. The internal fixation provided by the wires enhances the biomechanical environment, stimulating bone union. This application of intramedullary fixation is particularly relevant for fractures that have not responded adequately to conservative measures, offering a surgical intervention to address delayed or impaired healing [13].

**Figure 2:**
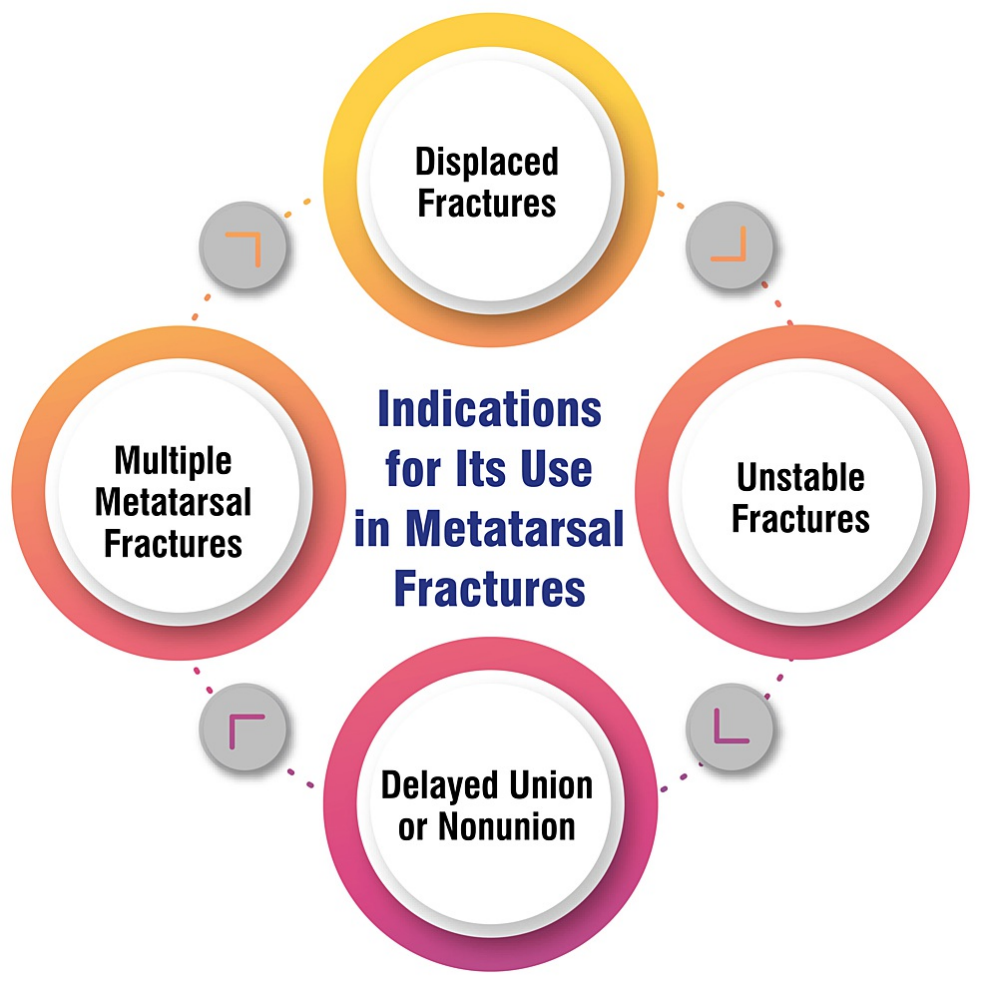
Indications for the use of internal fixation in metatarsal fractures The image is created by the corresponding author.

Multiple metatarsal fractures: In scenarios involving fractures affecting multiple metatarsals, intramedullary fixation offers a comprehensive and stable solution. Managing fractures in multiple metatarsals presents unique challenges due to the potential for increased instability and complexity. Intramedullary K-wires can be strategically placed to stabilize multiple fractures simultaneously. This approach minimizes the need for multiple incisions and external fixation devices, streamlining the surgical process. By providing stable internal fixation for each affected metatarsal, this method supports the coordinated healing of multiple fractures, contributing to a more efficient and integrated recovery process for the patient [9]. Figure 2 shows indications for its use in metatarsal fractures.

Surgical technique

Patient Selection

Fracture type and severity: Patient selection for intramedullary K-wire fixation initiates with a comprehensive evaluation of the metatarsal fracture, considering its type and severity. This technique is particularly well-suited for fractures that are displaced or unstable. Displacement involves a misalignment of the fractured bone ends, and instability implies a lack of structural support, which can be effectively addressed by intramedullary fixation. Tailoring the intervention to the specific fracture characteristics ensures that the chosen method aligns with the patient's unique needs, optimizing outcomes by promoting stability and anatomical restoration [9].

General health status: The patient's overall health status is a critical determinant in the decision-making process for intramedullary fixation. Evaluating comorbidities, nutritional status, and patient compliance with postoperative care regimens are essential considerations. Certain medical conditions or compromised health may influence the feasibility and safety of surgery. A thorough assessment helps identify potential risks and allows for developing a tailored surgical and postoperative care plan. This holistic approach ensures that the patient is physically prepared for the procedure and has the capacity for a successful recovery [14].

Bone quality: Adequate bone quality is a pivotal factor influencing the success of intramedullary fixation. Conditions such as osteoporosis, characterized by reduced bone density and strength, can impact the stability of the fixation. In cases where bone quality is compromised, alternative fixation methods may need to be considered. Assessing bone quality through preoperative imaging studies, such as dual-energy X-ray absorptiometry (DEXA) scans, assists surgeons in making informed decisions about the suitability of intramedullary fixation. This consideration emphasizes the importance of tailoring the surgical approach to the specific biomechanical characteristics of the patient's bones [15].

Patient compliance: Patient understanding and commitment to postoperative care are integral to successful intramedullary K-wire fixation. This involves adherence to instructions regarding weight-bearing restrictions, rehabilitation protocols, and follow-up appointments. Patients must comprehend the importance of actively participating in their recovery process, as non-compliance may jeopardize the efficacy of the intervention. Establishing clear communication channels between healthcare providers and patients facilitates informed decision-making, setting realistic expectations and ensuring a collaborative approach to achieving optimal outcomes. Patient compliance is a dynamic factor that significantly contributes to intramedullary fixation's overall success and effectiveness for metatarsal fractures [16].

Preoperative Planning

Imaging studies: Radiographic studies, such as X-rays, are pivotal in the preoperative assessment of metatarsal fractures. X-rays provide detailed images of the bone structure, allowing surgeons to evaluate the fracture pattern, displacement, and overall bone health. They offer essential information to guide decision-making, helping surgeons determine the most appropriate surgical intervention. In some cases, advanced imaging modalities such as CT scans may be employed where a more comprehensive understanding is required. CT scans provide detailed three-dimensional images, aiding in a more precise assessment of complex fractures, guiding surgical planning, and contributing to a thorough preoperative evaluation [17].

Fracture classification: Classifying metatarsal fractures according to established systems is a fundamental step in preoperative planning. Various classification systems exist, categorizing fractures based on location, pattern, and severity. This classification aids surgeons in understanding the specific characteristics of the fracture and guides the selection of an appropriate surgical approach. The classification helps predict the stability of the fracture, determines the likelihood of complications, and informs decisions regarding the optimal fixation strategy. By systematically categorizing fractures, surgeons can tailor their interventions to the unique features of each case, optimizing outcomes and streamlining the surgical process [18].

Surgical site marking: Precise identification of the surgical site and planning the entry points for K-wires are crucial aspects of preoperative preparation. Marking these locations on the patient's skin before surgery ensures accuracy. Surgeons strategically mark incision sites and entry points for wires based on preoperative imaging and fracture classification. This meticulous marking contributes to the efficiency and precision of the surgery, reducing the risk of errors and ensuring that the wires are placed in optimal positions. Surgical site marking serves as a visual guide for the surgical team, enhancing the overall safety and success of the intramedullary K-wire fixation procedure [19].

Anesthesia Considerations

Local anesthesia: Intramedullary K-wire fixation is frequently conducted under local anesthesia with sedation. This approach involves administering anesthesia directly to the surgical site, providing pain relief and facilitating patient comfort during the procedure. Local anesthesia minimizes the risks associated with general anesthesia, making it an attractive option for certain patients, especially those with specific medical conditions that may pose challenges under general anesthesia. Sedation is often added to induce a state of relaxation and ease any potential anxiety the patient may experience. This localized approach contributes to a quicker postoperative recovery, allowing for faster emergence from anesthesia and a potentially smoother transition to the postoperative phase [20].

General anesthesia: In specific cases, the nature of the procedure or considerations related to patient comfort may warrant general anesthesia for intramedullary K-wire fixation. General anesthesia induces a controlled state of unconsciousness, ensuring that the patient is entirely unaware and unresponsive throughout the surgery. This approach may be preferred for patients who may find local anesthesia insufficient for comfort or for procedures requiring complete immobility and relaxation. The selection of anesthesia is based on a careful evaluation of the patient's medical history, overall health status, and the preferences of the surgical team. While general anesthesia may pose some inherent risks, it is a valuable option for ensuring patient comfort and facilitating the successful completion of the surgical procedure. The choice between local and general anesthesia is a collaborative decision involving the surgical team and the patient, considering various factors to optimize the overall safety and efficacy of intramedullary K-wire fixation [21].

Step-by-Step Procedure of Intramedullary K-wire Fixation

The intramedullary K-wire fixation procedure follows a systematic approach. It begins with positioning the patient supine and ensuring a sterile field through draping. A small incision is made, and soft tissues are dissected to access the medullary canal without trauma. A guide wire is inserted under fluoroscopic guidance, followed by sequential K-wires for stabilizing fractures. Fluoroscopy confirms proper alignment. Wires are externally fixed, excess length is cut, and the incision is closed meticulously [22]. Postoperative care includes instructions on weight-bearing, rehabilitation, and follow-up appointments tailored to the specific case. The procedure is explained step by step in Table 1 [22].

**Table 1:** Step-by-step procedure of intramedullary K-wire fixation K-wire: Kirschner wire

Step	Procedure Description
1.	Patient Positioning: The patient is supine on the operating table, with the affected limb appropriately exposed and prepped.
2.	Sterile Draping: Ensure a sterile surgical field is maintained by adequately draping the limb and surrounding areas.
3.	Incision and Soft Tissue Handling: Make a small incision over the planned entry point and carefully dissect soft tissues to access the medullary canal without causing unnecessary trauma.
4.	Guide Wire Placement: Carefully insert a guide wire into the medullary canal under fluoroscopic guidance, ensuring accurate placement and alignment.
5.	Sequential K-wire Insertion: Following the guide wire, advance sequential K-wires of appropriate diameter to stabilize the fractured segments, avoiding excessive penetration.
6.	Fluoroscopic Confirmation: Throughout the procedure, use fluoroscopy to confirm proper wire placement, alignment, and fracture reduction.
7.	Wire Fixation and Cut: Once all wires are in place, externally fix them and cut excess length to prevent soft tissue irritation.
8.	Wound Closure: Meticulously close the incision, apply dressings, and perform postoperative imaging to confirm the adequacy of reduction and fixation.
9.	Postoperative Care: Depending on the specific case, provide postoperative care instructions, including weight-bearing restrictions, rehabilitation protocols, and follow-up appointments.

Treatment outcomes

Review of Clinical Studies and Trials

The study "Retrospective comparative analysis between intramedullary Kirschner wire fixation and conservative treatment for displaced 5^th^ metacarpal neck fractures" found that conservative treatment has comparable outcomes to intramedullary K-wire fixation for displaced 5^th ^metacarpal neck fractures [23]. "Intramedullary resorbable fixation system versus K-wire for the treatment of lesser toe deformities", a clinical trial, compares the fixation of digital arthrodesis with a percutaneous K-wire versus an intramedullary fixation with a resorbable polylactic acid needle [24]. The study aims to determine the effectiveness and safety of the intramedullary resorbable fixation system compared to the traditional K-wire fixation.

Another study "Intramedullary single-Kirschner-wire fixation in displaced fractures of the fifth metacarpal neck (boxer's neck)" reports that the procedure provides sound fracture reduction and stabilization, with satisfactory outcomes and very low disabilities of the arm, shoulder, and hand (DASH) scores [25]. The study "Closed antegrade/retrograde intramedullary fixation of central metatarsal fractures: surgical technique and clinical outcomes" demonstrates that intramedullary fixation with K-wires is a surgical option for managing fractures of the shaft and neck of central metatarsals [26].

The study "Percutaneous fixation of metatarsals fractures with Kirchner’s wire, comparison of antegrade and retrograde technique" compares the antegrade and retrograde techniques for K-wire fixation of metatarsal fractures. The study found that the antegrade technique (EAK) presented a lower rate of metatarsalgia and shorter rest time compared to the retrograde technique (ERK) [27]. These studies suggest that intramedullary K-wire fixation is a viable treatment option for various orthopedic conditions, including metatarsal fractures. However, the fixation technique and the need for surgery should be determined case-by-case, considering the patient's condition, age, and activity level.

Comparison With Other Treatment Modalities

A retrospective comparative analysis between intramedullary K-wire fixation and conservative treatment for displaced 5^th^ metacarpal neck fractures found that both modalities have good outcomes [23]. A study comparing radiologic and functional outcomes of two different K-wire fixation methods and immobilization techniques of extra- and intra-articular distal radius fractures found that any technique maintaining the radial length may attain better functional results [23]. A study on treating two or more metatarsal fractures found that surgical treatment is typically done by percutaneous or open pinning. Still, this fixation method has been criticized by some authors [28].

A clinical trial is ongoing to compare the fixation of digital arthrodesis with percutaneous K-wire versus intramedullary fixation with a resorbable polylactic acid needle for the treatment of lesser toe deformities [24]. A study comparing open intramedullary K-wire versus screw and plate fixation for unstable forearm fractures in children found that both modalities have good outcomes [29]. These studies suggest that intramedullary K-wire fixation is a viable treatment option for various orthopedic conditions, and it may have comparable outcomes to other treatment modalities. However, the choice of treatment modality should be determined on a case-by-case basis, considering the patient's specific condition, age, and activity level.

Complications and Their Management

Intramedullary K-wire fixation is generally considered a reliable treatment for various fractures, but it is not without potential complications. Complications associated with this fixation method may include infection, nonunion, malunion, hardware failure, and damage to surrounding structures [23,30]. In the case of infection, it may be managed with appropriate antibiotics and, in some cases, surgical debridement. Nonunion and malunion may require revision surgery to achieve proper healing and alignment. Hardware failure, such as migration or breakage of the K-wire, may necessitate removal or revision of the hardware. Damage to surrounding structures, such as nerves or blood vessels, is a rare but severe complication that may require further surgical intervention [23,30].

Long-Term Outcomes and Patient Satisfaction

Long-term outcomes and patient satisfaction following intramedullary K-wire fixation have been generally favorable. For example, a study on displaced fractures of the 5^th^ metacarpal neck treated with intramedullary single K-wire fixation reported satisfactory outcomes and very low DASH scores [25]. However, long-term outcomes and patient satisfaction may vary depending on the specific fracture, patient characteristics, and any complications. Patients need to be followed up regularly by their healthcare provider to monitor the healing process, function, and any potential long-term issues that may arise.

Advances and innovations

Emerging Technologies in Intramedullary Fixation

Biodegradable implants: The advancement of biodegradable materials for intramedullary fixation represents a significant innovation in orthopedic surgery. Biodegradable implants gradually break down and get absorbed by the body over time, eliminating the need for a second surgery for removal. This is particularly beneficial compared to traditional permanent implants, as it reduces the risk of long-term complications associated with hardware, such as irritation or infection. Using biodegradable implants streamlines the overall treatment process, enhances patient comfort, and potentially lowers healthcare costs by eliminating the need for secondary removal surgery [31].

Smart implants: Integrating sensors and monitoring devices into intramedullary implants introduces the concept of innovative implants. These devices enable real-time assessment of the healing progress within the bone. By transmitting data to healthcare providers, innovative implants facilitate proactive intervention in case of delayed healing or other complications. This real-time monitoring enhances the ability to tailor postoperative care based on individual patient responses, promoting personalized medicine in orthopedic interventions. Incorporating innovative technology in implants represents a promising avenue for improving patient outcomes through timely and targeted interventions [32].

3D printing technology: Using 3D printing technology in intramedullary fixation allows customized implants tailored to each patient's unique anatomy. This personalized approach enhances the precision of implant placement, ensuring an optimal fit and alignment. Customized implants can address variations in bone morphology and fracture patterns, potentially reducing the risk of complications associated with standardized implants. 3D printing technology also offers flexibility in material selection, allowing for the creation of implants with specific mechanical properties suited to individual patient needs. This innovation marks a significant step towards advancing the accuracy and efficacy of intramedullary fixation [33].

**Figure 3:**
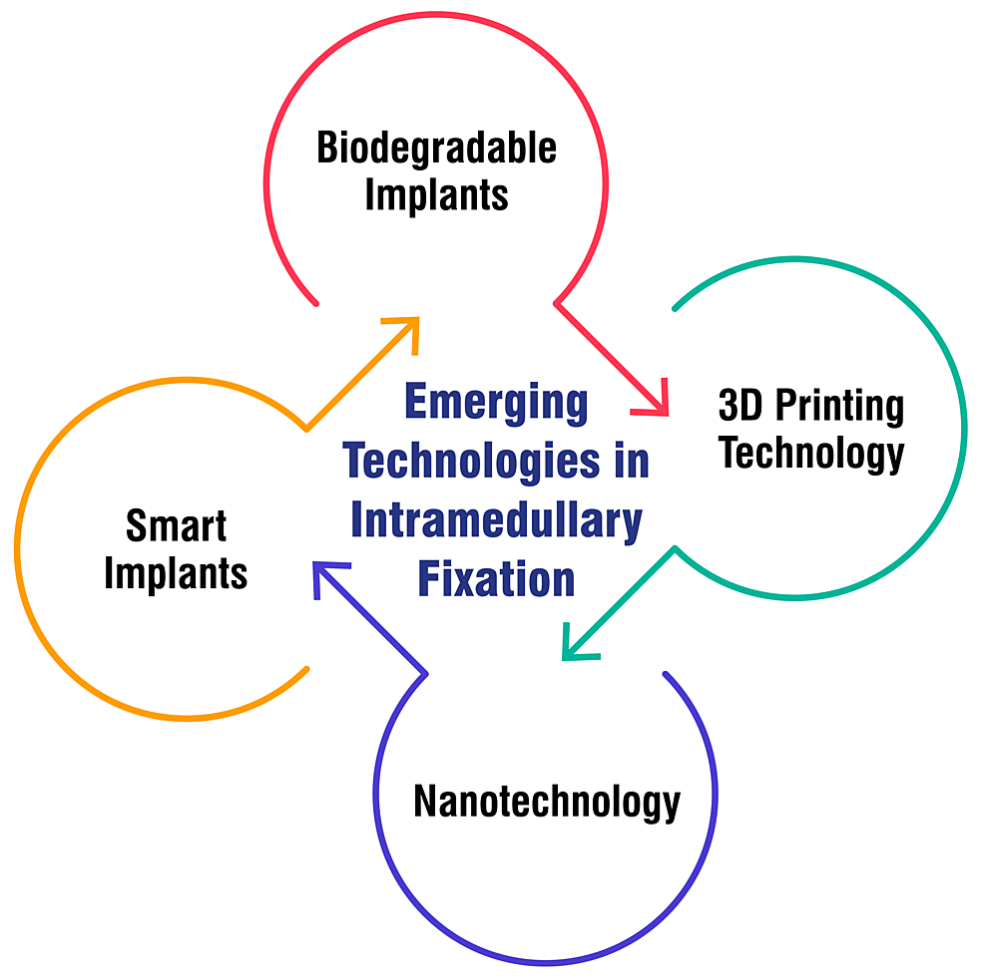
Emerging technologies in intramedullary fixation The image is created by the corresponding author.

Nanotechnology: Incorporating nanotechnology in intramedullary fixation focuses on enhancing the material properties of implants at the nanoscale. Nanomaterials exhibit unique characteristics, including improved strength, biocompatibility, and reduced inflammatory responses. These properties contribute to more favorable long-term results by minimizing the risk of implant-related complications. The application of nanotechnology in intramedullary fixation aligns to optimize the biophysical interaction between implants and the surrounding bone tissue. As the field continues to evolve, nanotechnology can further enhance the safety and performance of intramedullary implants in orthopedic surgery [34]. Figure 3 shows emerging technologies in intramedullary fixation.

Ongoing Research and Potential Future Developments

Biological augmentation: Ongoing research investigates the potential of biological augmentation combined with intramedullary fixation to enhance bone healing. Growth factors, stem cells, or other regenerative therapies may be applied to the fracture site, promoting a more robust and accelerated recovery. By harnessing the body's natural healing mechanisms, biological augmentation aims to optimize the environment for bone regeneration, potentially reducing healing time and enhancing the overall success of intramedullary fixation [35].

Virtual surgical planning (VSP): Adopting VSP allows surgeons to conduct meticulous preoperative simulations. By virtually assessing fracture patterns, planning implant placement, and optimizing the entire surgical procedure, VSP contributes to improved intraoperative precision. Surgeons can anticipate challenges, refine their approach, and potentially reduce the risk of intraoperative complications. This technology enhances planning, providing a valuable tool for optimizing outcomes in intramedullary fixation procedures [36].

Improved imaging modalities: Advances in imaging technologies, such as higher resolution CT scans and advanced MRI techniques, contribute to more accurate preoperative planning and intraoperative guidance in intramedullary fixation procedures. These enhanced imaging modalities give surgeons more precise insights into fracture morphology, facilitating better decision-making regarding implant selection and placement. Improved visualization of bone quality and surrounding structures contributes to increased surgical precision and may improve patient outcomes [37].

Customized implant materials: Ongoing exploration of novel implant materials, including bioresorbable polymers and composite materials, addresses specific challenges associated with traditional metallic implants. Customized materials with tailored mechanical properties may offer improved biocompatibility and long-term performance. The development of implants that closely mimic the biomechanical properties of natural bone could potentially enhance the success and longevity of intramedullary fixation while minimizing the risk of complications associated with traditional materials [38].

**Figure 4:**
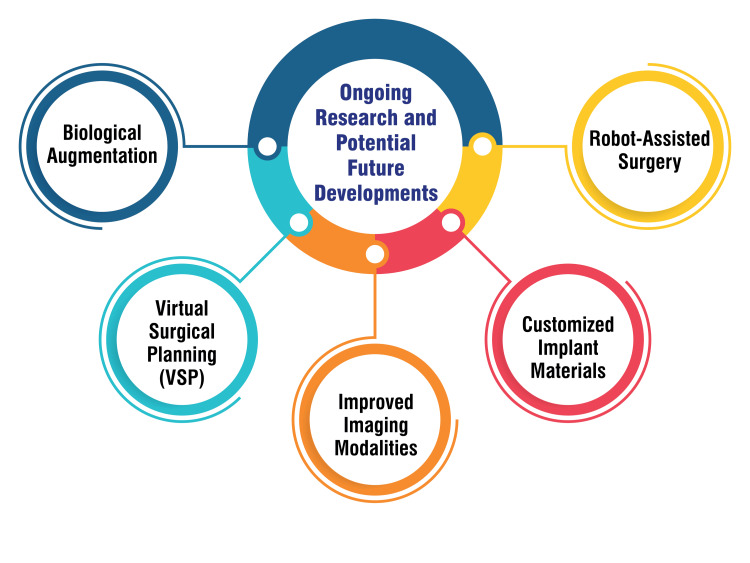
Ongoing research and potential future developments The image is created by the corresponding author.

Robot-assisted surgery: Integrating robotics in orthopedic surgery, including intramedullary fixation procedures, is a promising area of research. Robot-assisted surgery enhances precision by providing surgeons with advanced planning and execution tools. This technology reduces variability in surgical techniques and may contribute to shorter surgical times. The robotic assistance in implant placement aims to optimize accuracy and improve the reproducibility of surgical outcomes. As this field continues to evolve, robot-assisted surgery can further enhance the overall safety and efficacy of intramedullary fixation procedures [39]. Figure 4 shows ongoing research and potential future developments.

Considerations for rehabilitation

Postoperative Care and Rehabilitation Protocols

Early mobilization: Encouraging early mobilization is a cornerstone of the rehabilitation process following intramedullary K-wire fixation. Early mobilization is crucial for preventing joint stiffness and muscle atrophy within the limits defined by weight-bearing restrictions. Patients are guided to initiate controlled movements when the surgical team deems it appropriate. This proactive approach to mobilization contributes to improved joint flexibility, muscle strength, and overall functional recovery. Early mobilization is tailored to the individual patient, considering the fracture's specific characteristics and the fixation's stability [40].

Weight-bearing guidelines: Clear and specific weight-bearing guidelines are communicated to the patient based on the fixation's stability and the metatarsal fracture's characteristics. These guidelines dictate the extent to which the patient can bear weight on the affected foot during different phases of the recovery process. Initially, partial weight-bearing may be recommended to protect the healing bone, gradually progressing to full weight-bearing as the fracture consolidates and demonstrates sufficient stability. Strict adherence to these weight-bearing guidelines is crucial for preventing complications and ensuring the success of the intramedullary fixation [41].

Wound care: Strict adherence to wound care protocols is essential to prevent infections and complications at the incision site. Patients are provided with guidelines on keeping the incision clean and dry. Regular follow-up appointments with healthcare providers allow for ongoing monitoring and assessment of wound healing progress. Any signs of infection, such as redness, swelling, or discharge, are promptly addressed to minimize the risk of complications. Wound care is a critical component of postoperative management, ensuring the integrity of the surgical site and facilitating an uneventful recovery [42].

Pain management: Effective pain management strategies are implemented to address postoperative discomfort. This may involve using analgesic medications, anti-inflammatory drugs, and cold compresses. Patients are educated on the proper administration of pain medications and encouraged to communicate any concerns or changes in pain levels to the healthcare team. Monitoring for signs of excessive pain or complications is paramount to tailor pain management strategies to individual patient needs and enhance overall postoperative comfort [43].

Orthotic devices: Depending on the nature of the metatarsal fracture and the surgical technique employed, orthotic devices such as walking boots or braces may be prescribed. These devices provide additional support during the initial stages of rehabilitation, helping to protect the healing bone and maintain stability. Orthotic devices are often used with weight-bearing guidelines and are gradually phased out as the patient progresses through the rehabilitation process. The prescription and proper use of orthotic devices contribute to the overall success of intramedullary K-wire fixation by supporting the healing process and preventing complications during the recovery period [44].

Expected Recovery Timeline

Early phase (0-6 weeks): During the early phase of rehabilitation (0-6 weeks), the primary focus is on facilitating wound healing, managing pain, and initiating the gradual reintroduction of weight-bearing activities. Careful monitoring of the surgical site ensures the incision heals properly, minimizing the risk of infection or other complications. Pain management strategies, including medications and cold compresses, aim to enhance patient comfort during the initial recovery period. Weight-bearing restrictions are followed, as determined by the stability of the fixation and fracture healing progress. Follow-up X-rays may be performed to assess the early stages of fracture healing and guide decisions on rehabilitation progression [45].

Intermediate phase (6 weeks-3 months): In the intermediate phase (6 weeks-3 months), a more structured rehabilitation program is introduced as fracture healing progresses. Physical therapy becomes a central component, incorporating exercises to improve range of motion, strength, and proprioception. The focus is on gradually increasing the intensity and complexity of exercises to enhance the overall functionality of the foot. Based on the clinical assessment and imaging findings, weight-bearing restrictions may be gradually lifted. This phase aims to restore optimal biomechanics and prepare the patient for more advanced activities in the subsequent stages of rehabilitation [46].

Advanced phase (3 months and beyond): The advanced phase (3 months and beyond) marks the transition toward restoring normal function and strength. At this stage, weight-bearing is typically unrestricted, and patients engage in activities to enhance overall foot function. The rehabilitation program may include specific exercises targeting balance, coordination, and agility. Activities that simulate daily tasks or sports-related movements may be incorporated to promote functional recovery. Continued monitoring ensures that the patient achieves maximal functional recovery, and adjustments to the rehabilitation program are made based on individual progress. Gradual return to normal activities, including sports and recreational pursuits, is encouraged to prevent reinjury and promote long-term foot health [47].

Physical Therapy Recommendations

Range of motion exercises: Controlled range of motion exercises are crucial in preventing joint stiffness and maintaining flexibility in the foot and ankle. These exercises are introduced early in rehabilitation and adapted based on the patient's progress. The goal is to gradually restore and improve the normal range of motion in the affected joints, promoting optimal biomechanics and preventing long-term restrictions in movement [48].

Strengthening exercises: Targeted strengthening exercises focus on both intrinsic and extrinsic muscles of the foot and lower leg. Strengthening these muscles is essential for stabilizing the foot, improving balance, and preventing muscle atrophy that may have occurred during the recovery period. Progressive resistance exercises are employed to challenge the muscles and gradually build strength, contributing to the overall functional recovery of the foot [49].

Proprioception training: Proprioception exercises enhance the patient's awareness of foot position and movement. These exercises promote better balance and proprioceptive control, reducing the risk of recurrent injuries. Proprioception training often involves activities that challenge balance and coordination, helping the patient regain confidence in weight-bearing and dynamic movements [50].

Gait training: Gait training is a critical component of rehabilitation to restore standard walking patterns. This involves assessing and correcting any abnormalities in the patient's gait that may have developed during recovery. Gait training addresses uneven weight distribution altered stride length, or compensatory movements. By optimizing gait mechanics, patients can safely transition back to normal walking and reduce the risk of developing gait-related complications [51].

Functional activities: The gradual incorporation of functional activities, such as stair climbing, jumping, and agility drills, ensures the patient can safely resume normal daily activities and recreational pursuits. These activities mimic real-life movements and challenges, progressively allowing patients to reintegrate into their routines. The introduction of functional activities is tailored to the patient's progress and serves as a bridge between basic rehabilitation exercises and more dynamic, real-world tasks [52].

Patient education: Educating patients about the importance of adherence to rehabilitation protocols is integral to long-term success. Providing information on recognizing warning signs of complications, emphasizing the need for regular follow-up appointments, and offering guidance on maintaining overall foot health empowers patients to participate actively in their recovery. Patient education contributes to informed decision-making, reduces the risk of setbacks, and promotes a proactive approach to long-term foot care [53]

## Conclusions

In conclusion, the review underscores the efficacy and advantages of intramedullary K-wire fixation in addressing metatarsal fractures. The technique's inherent stability and minimally invasive nature make it a compelling option for displaced and unstable fractures, promoting anatomical alignment and potentially enabling early mobilization. Critical recommendations for clinical practice include meticulous patient selection, comprehensive preoperative planning, and a strong emphasis on postoperative care and rehabilitation. Multidisciplinary collaboration among orthopedic surgeons, anesthesiologists, and rehabilitation specialists is encouraged for a holistic approach to patient management. Despite the promising outcomes, future research avenues should explore comparative studies with other treatments, long-term follow-up assessments, advanced imaging techniques, biomechanical studies, and strategies for complication management. As the field evolves, these insights aim to guide clinicians and researchers in refining practices and expanding the scope of intramedullary K-wire fixation in metatarsal fracture care.
